# RNA3DCNN: Local and global quality assessments of RNA 3D structures using 3D deep convolutional neural networks

**DOI:** 10.1371/journal.pcbi.1006514

**Published:** 2018-11-27

**Authors:** Jun Li, Wei Zhu, Jun Wang, Wenfei Li, Sheng Gong, Jian Zhang, Wei Wang

**Affiliations:** 1 National Laboratory of Solid State Microstructures, School of Physics, Collaborative Innovation Center of Advanced Microstructures, Nanjing University, Nanjing, China; 2 State Key Laboratory for Novel Software Technology, Nanjing University, Nanjing, China; 3 Department of Pharmaceutics, Nanjing General Hospital, Nanjing University Medical School, Nanjing, China; University of Missouri, UNITED STATES

## Abstract

Quality assessment is essential for the computational prediction and design of RNA tertiary structures. To date, several knowledge-based statistical potentials have been proposed and proved to be effective in identifying native and near-native RNA structures. All these potentials are based on the inverse Boltzmann formula, while differing in the choice of the geometrical descriptor, reference state, and training dataset. Via an approach that diverges completely from the conventional statistical potentials, our work explored the power of a 3D convolutional neural network (CNN)-based approach as a quality evaluator for RNA 3D structures, which used a 3D grid representation of the structure as input without extracting features manually. The RNA structures were evaluated by examining each nucleotide, so our method can also provide local quality assessment. Two sets of training samples were built. The first one included 1 million samples generated by high-temperature molecular dynamics (MD) simulations and the second one included 1 million samples generated by Monte Carlo (MC) structure prediction. Both MD and MC procedures were performed for a non-redundant set of 414 RNAs. For two training datasets (one including only MD training samples and the other including both MD and MC training samples), we trained two neural networks, named RNA3DCNN_MD and RNA3DCNN_MDMC, respectively. The former is suitable for assessing near-native structures, while the latter is suitable for assessing structures covering large structural space. We tested the performance of our method and made comparisons with four other traditional scoring functions. On two of three test datasets, our method performed similarly to the state-of-the-art traditional scoring function, and on the third test dataset, our method was far superior to other scoring functions. Our method can be downloaded from https://github.com/lijunRNA/RNA3DCNN.

## Author summary

RNA is an important and versatile macromolecule participating in various biological processes. In addition to experimental approaches, the computational prediction of RNA 3D structures is an alternative and important source of obtaining structural information and insights into their functions. An important part of these computational prediction approaches is structural quality assessment. For this purpose, we developed a 3D CNN-based approach named RNA3DCNN. This approach uses raw atom distributions in 3D space as the input of neural networks and the output is an RMSD-based nucleotide unfitness score for each nucleotide in an RNA molecule, thus making it possible to evaluate local structural quality. Here, we tested and made comparisons with four other traditional scoring functions on three test datasets from different sources.

## Introduction

RNA molecules consist of unbranched chains of ribonucleotides, which have various essential roles in coding, decoding, regulation, expression of genes, and cancer-related networks via the maintenance of stable and specific 3D structures [1–5]. Therefore, their 3D structural information would help fully appreciate their functions. In this context, experiments such as X-ray crystallography, nuclear magnetic resonance (NMR) spectroscopy, and cryoelectron microscopy are the most reliable methods of determining RNA 3D structures, but they are costly, time-consuming, or technically challenging due to the physical and chemical nature of RNAs. As a result, many computational methods have been developed to predict RNA tertiary structures [6–32]. These methods usually have a generator producing a large set of structural candidates and a discriminator evaluating these generated candidates. A good generator should be able to produce structural candidates as close to native structures as possible, and a good discriminator should be able to recognize the best candidates. Moreover, a discriminator can direct generator searching structural space in heuristic prediction methods. For protein or RNA tertiary structure prediction, a discriminator generally refers to a free energy function, a knowledge-based statistical potential, or a scoring function.

Several statistical potentials have been developed to evaluate RNA 3D structures, such as RASP [33], RNA KB potentials [34], 3dRNAscore [35] and the Rosetta energy function [9, 16]. Generally, these potentials are proportional to the logarithm of the frequencies of occurrence of atom pairs, angles, or dihedral angles based on the inverse Boltzmann formula. The all-atom version of RASP defines 23 atom types, uses distance-dependent geometrical descriptions for atom pairs with a bin width of 1 Å, and is derived from a non-redundant set of 85 RNA structures. The all-atom version of RNA KB potential defines 85 atom types, also uses distance-dependent geometrical descriptions for atom pairs, and is derived from 77 selected representative RNA structures. Moreover, RNA KB potentials are fully differentiable and are likely useful for structure refinement and molecular dynamics simulations. 3dRNAscore also defines 85 atom types and uses distance-dependent geometrical descriptions for atom pairs with a bin width of 0.15 Å, and is derived from an elaborately compiled non-redundant dataset of 317 structures. In addition to distance-dependent geometrical descriptions, 3dRNAscore uses seven RNA dihedral angles to construct the statistical potentials with a bin width of 4.5°, and the final output potentials are equal to the sum of the two energy terms with an optimized weight. The Rosetta energy function has two versions: one for low resolution and the other for high resolution. The low-resolution knowledge-based energy function explicitly describing the base-pairing and base-stacking geometries guides the Monte Carlo sampling process in Rosetta, while the more detailed and precise high-resolution all-atom energy function can refine the sampled models and yield more realistic structures with cleaner hydrogen bonds and fewer clashes. As the paper on 3dRNAscore reported, 3dRNAscore is the best among these four scoring functions. Overall, the choices of the geometrical descriptors and the reference states in the scoring functions can affect their performance significantly, and the optimization of the parameters also influences this.

Recently, we have witnessed astonishing advances in machine learning as a tool to detect, characterize, recognize, classify, or generate complex data and its rapid applications in a broad range of fields, from image classification, face detection, auto driving, financial analysis, disease diagnosis [36], playing chess or games [37, 38], and solving biological problems [39–42], to even quantum physics [43–45]. Even this list is incomplete, and has the potential to be extended further in the future. Therefore, we expect that machine learning methods will be able to help evaluate the structural candidates generated in the process of RNA tertiary structure prediction. Inspired by the successful application of 2D convolutional neural networks (CNNs) in image classification, we believe that 3D CNNs are a promising solution in that RNA molecules can be treated as a 3D image. Compared with other machine learning methods employing conventional hand-engineered features as input, 3D CNNs can directly use a 3D grid representation of the structure as input without extracting features manually. 3D CNNs have been applied to computational biology problems such as the scoring of protein–ligand poses [46, 47], prediction of ligand–binding protein pockets [48], prediction of the effect of protein mutations [49], quality assessment of protein folds [50], and prediction of protein–ligand binding affinity [51].

Here, we report our work on developing two new scoring functions for RNA 3D structures based on 3D deep CNNs, which we name RNA3DCNN_MD and RNA3DCNN_MDMC, respectively. Our scoring functions enable both local and global quality assessments. To our knowledge, this is the first paper to describe the use of 3D deep CNNs to assess the quality of RNA 3D structures. We also tested the performance of our approaches and made comparisons with the four aforementioned energy functions.

## Materials and methods

In our work, the evaluation of an RNA structure is divided into the assessment of each nucleotide. The nucleotide to be evaluated and its surrounding atoms are treated as a 3D image that is fed into the 3D CNNs, and the output is an RMSD-based nucleotide unfitness score characterizing how poorly the nucleotide fits into its surrounding environment. The philosophy of our method is that though the global structures of the RNAs differ from each other, the local building blocks should recur frequently, such as helix, bulge, internal loops, and junctions, and thus we used a cube of local atoms as our CNN input rather than the whole structures. The well-trained networks can give an unfitness score to each nucleotide, and the evaluation score for a structure is equal to the sum of the unfitness scores of all of its nucleotides. In the following subsections, we elaborate on the definition of the surrounding environment, the input and output, the architecture and configurations of the 3D CNNs, the training processes, the training and test datasets.

### Environment surrounding a nucleotide

The environment surrounding a nucleotide refers to its neighboring. To determine the neighboring atoms of a nucleotide, a local Cartesian coordinate system is specified first by its atoms C1’, O5’, C5’, and N1 for pyrimidine or N9 for purine. Specifically, the origin of the local coordinate system is located at the position of atom C1’. The x-, y-, and z-axes of the local coordinate system, denoted as **x**, **y**, and **z**, respectively, are decided according to Eqs 1–6 where **r**_**C1′**_, **r**_**O5′**_, **r**_**C5′**_ and **r**_**N**_ stand for the vectors pointing from the origin in the global coordinate system to the atoms C1’, O5’, C5’, and N1 or N9, respectively.

$$\begin{array}{c} x=r_{N}-r_{C1\prime } \end{array}$$ (1)

$$\begin{array}{c} x=\frac{x}{∥x∥} \end{array}$$ (2)

$$\begin{array}{c} y=\frac{r_{O5\prime }+r_{C5\prime }}{2}-r_{C1\prime } \end{array}$$ (3)

$$\begin{array}{c} z=x\times y \end{array}$$ (4)

$$\begin{array}{c} z=\frac{z}{∥z∥} \end{array}$$ (5)

$$\begin{array}{c} y=z\times x \end{array}$$ (6)

**Fig 1:**
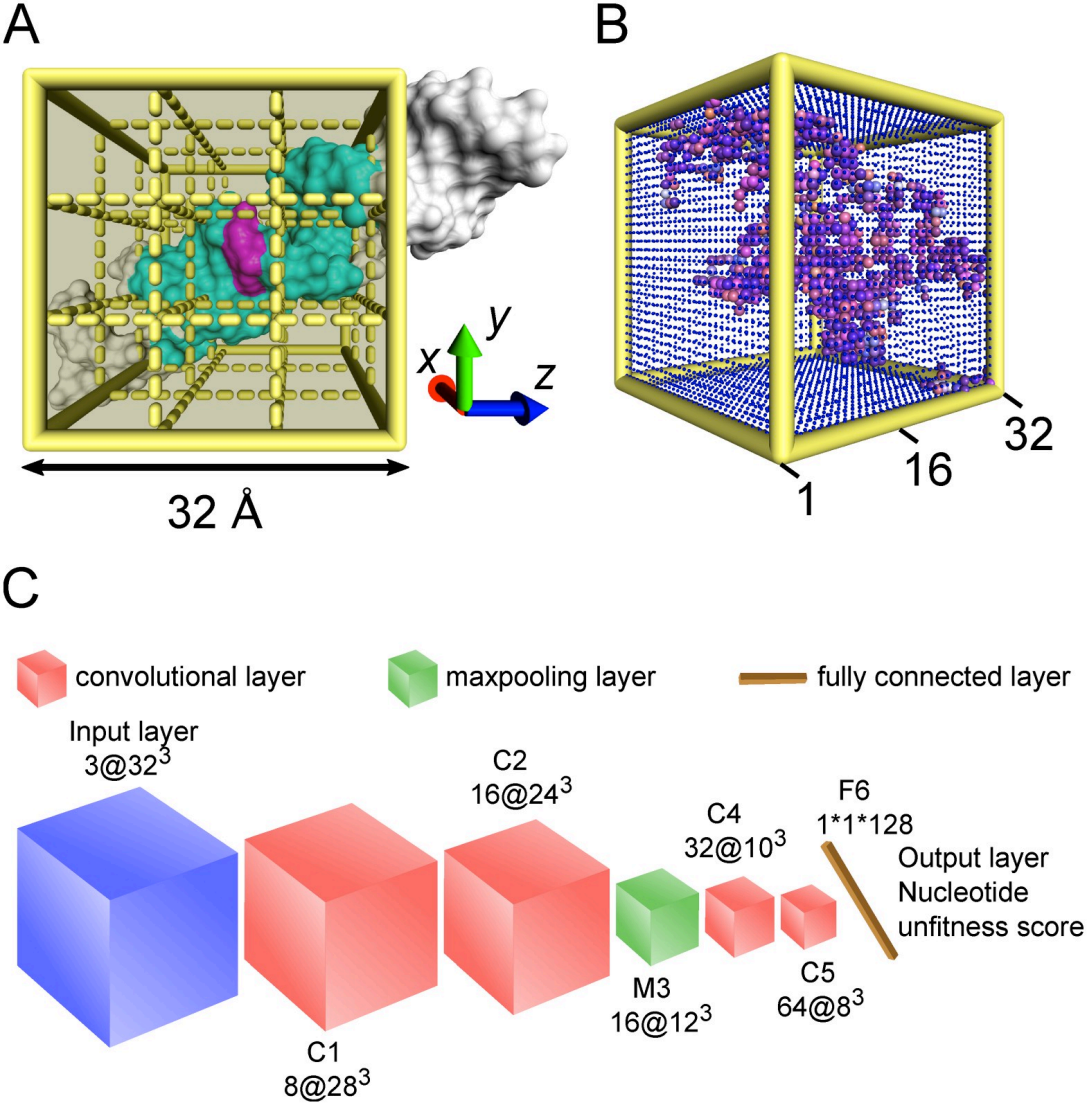
The input and architecture of the 3D deep CNN in this work. (A) Surrounding environment extraction. The magenta color depicts the nucleotide under assessment. Based on the assessed nucleotide, a local Cartesian coordinate system xyz is determined and accordingly a cube with sides of length 32 Å is drawn as yellow solid lines. See the section Environment surrounding a nucleotide for details. The surrounding environment is precisely defined by the cyan-colored atoms within the box and the gray atoms beyond the box do not influence the assessment of the magenta nucleotide. (B) RNA voxelizations. The box in (A) is partitioned into 32 × 32 × 32 grid boxes drawn as yellow dashed lines, but for visual convenience, only 3 × 3 × 3 grid boxes are drawn. Each grid box resembles a voxel of three channels representing atomic occupation number, mass, and charge within the grid box. As shown in (B), an assessed nucleotide and its surrounding environment are voxelated into a 3D image of 32 × 32 × 32 voxels. The RGB channels are mapped to the three channels. The large and small spheres represent the voxels occupied by atoms and nothing, respectively, while the size difference of the spheres does not represent anything but is only for visual comparison. (C) CNN architecture. The network is arranged in the order of input layer, convolutional layer C1, convolutional layer C2, maxpooling layer M3, convolutional layer C4, convolutional layer C5, and output layer. Blue, red, and green cubes represent the input layer, convolutional layer, and maxpooling layer, respectively. The yellow stick represents the output layer. *m*@*n*^3^ means that there are *m* channels and *n*^3^ voxels.

The environment surrounding a nucleotide consists of the atoms whose absolute values of x, y, and z coordinates are less than a certain threshold. Here, the threshold is set to 16 Å, which means that the environment surrounding a nucleotide contains the atoms within a cube of length 32 Å centered at this very nucleotide, as shown in Fig 1A.

### Input of CNN: A colorful 3D image

For a colorful 2D image, the input of a 2D CNN is an array of pixels of RGB channels. Similarly, in our work, the nucleotide and its surrounding environment are transformed into a 3D image consisting of an array of voxels. As shown in Fig 1A, the box of size 32 × 32 × 32 Å is partitioned into 32 × 32 × 32 grid boxes. Each grid box represents a voxel of three channels and its values are calculated by the accumulations of the occupation number, mass, or charge of the atoms in the grid box. The mass and charge information of each type of atoms is listed in S1 Table. After transformation, the input of the 3D CNN is a colorful 3D image of 32 × 32 × 32 voxels with three channels corresponding to RGB channels presented in Fig 1B. Practically, each channel is normalized to [0, 1] by min-max scaling.

### Output of CNN: Nucleotide unfitness score

The output of our CNN is the nucleotide unfitness score characterizing how poorly a nucleotide fits into its surroundings. For a nucleotide, its unfitness score is equal to the RMSD of its surroundings plus the RMSD of itself after optimal superposition between its conformations in the native structure and the assessed structure. The latter RMSD is generally very small, but the former varies in a large range. Nucleotides with smaller unfitness scores are in a conformation closer to the native conformation, and a score of 0 means that the nucleotide fits into its surrounding environment perfectly and is in its native conformation. Practically, the nucleotide unfitness score is normalized to [0, 1] by min-max scaling. For the global quality assessment, the unfitness scores of all nucleotides are accumulated.

### The architecture of the CNN

Fig 1C exhibits the architecture of our CNN, a small VGG-like network [52] containing a stack of convolutional layers, a maxpooling layer, a fully connected layer, and 4,282,801 parameters in total. VGGNet is a famous image classification CNN. It is a very deep network and uses 19 weight layers, consisting of 16 convolutional layers stacked on each other and three fully-connected layers. The input image size 224 × 224 in VGGNet is much larger than our input size 32 × 32 × 32 in terms of the side length, and thus we used a smaller architecture.

There are only four 3D convolutional layers in our neural network. The numbers of filters in each convolutional layer are 8, 16, 32, and 64, and the receptive fields of the filters in the first two convolutional layers and in the last two convolutional layers are 5 × 5 × 5 voxels and 3 × 3 × 3 voxels, respectively. The convolution stride is set to one voxel. No spatial padding is implemented in the convolutional layers. Moreover, a max-pooling layer of stride 2 is placed following the first two consecutive convolutional layers.

Subsequently, one fully connected layer with 128 hidden units is stacked after the convolutional layers. The final output layer is a single number, namely, the unfitness score. All units in hidden layers are activated by the ReLU nonlinear function, while the output layer is linearly activated.

### CNN training

The neural network was trained to reduce the mean squared error (MSE) between the true and predicted unfitness scores. A back-propagation-based mini-batch gradient descent optimization algorithm was used to optimize the parameters in the network. Batch size was set to 128. The training was regularized by dropout regularization for the second, fourth convolutional layers, and the fully connected layer with a dropout ratio of 0.2. The Glorot uniform initializer was used to initialize the network weights. The learning rate was initially set to 0.05, and then decreased by half whenever the MSE of the validation dataset stopped improving for five epochs. The training process stopped when the learning rate decreased to 0.0015625. Our 3D CNN was implemented using the python deep learning library Keras [53], with Theano library as the backend.

### Training dataset

To construct the training dataset, first a list of 619 RNAs was downloaded with the search options “RNA Only” and “Non Redundant RNA Structures” from the NDB website http://ndbserver.rutgers.edu/, which means that our training dataset includes RNA-only structures and the RNAs are non-redundant in both sequence and geometry. Second, the RNAs with an X-ray resolution >3.5 Å were removed from the list above. Finally, the RNAs in the test dataset were removed and the RNAs in the equivalence classes with the test dataset were also removed. “Structures that are provisionally redundant based on sequence similarity and also geometrical similarity are grouped into one equivalence class,” as Leontis et al. defined [54]. Thus, 414 native RNAs were left to construct the training dataset. According to their length, the 414 RNAs were randomly divided into two groups, namely, 332 RNAs for training and 82 RNAs for validation in the CNN training process. Practically, the training samples were generated in two ways, namely, by MD and MC methods elaborated as follows.

#### Training samples generated by MD methods

For each of the 414 RNAs, a 40-ns simulated annealing molecular dynamics simulation was run, with temperature gradually rising from 300 to 600 K by using the software Gromacs. According to their RMSDs to the native structure, 300 structures were randomly picked out of each trajectory for each RNA. Each nucleotide and its surrounding environment were treated as one sample. In total, 1 million training samples and 0.2 million validation samples were extracted from the 332 × 300 and 82 × 300 structures randomly based on their nucleotide unfitness scores, respectively. The training samples were used to fit the parameters in the neural network and the validation samples were used to determine when to decrease the learning rate and to choose the final neural network.

#### Training samples generated by MC methods

We used a macromolecular structure modeling software Rosetta to sample RNA 3D structures based on a fragment assembly method, which is a MC process guided by a low-resolution knowledge-based energy function [9, 16]. The models were further refined in an all-atom potential to yield more realistic structures. For each of the 414 RNAs, we fed the sequence and secondary structural information to Rosetta. The predicted models were clustered and 300 structures were picked out for each RNA. And like in MD methods, there were 1 million training samples and 0.2 million validation samples extracted from the 332 × 300 and 82 × 300 structures randomly based on their nucleotide unfitness scores, respectively.

### Test dataset

To evaluate our CNN-based scoring function and make comparisons with the traditional statistical potentials, three test datasets were collected from different sources.

Test dataset I comes from the RASP paper [33] which is generated by the MODELLER computer program from the native structures of 85 non-redundant RNAs given a set of Gaussian restraints for dihedral angles and atom distances, and contains 500 structural decoys for each of the 85 RNAs. The RMSDs are in different ranges for these RNAs. The narrowest are from 0 to 3.5 Å, the broadest are from 0 to 13 Å, and the RMSDs of most decoys are less than 10 Å. This dataset can be downloaded from http://melolab.org/supmat/RNApot/Sup._Data.html.

Test dataset II comes from the KB paper [34], which is generated by both position-restrained dynamics and REMD simulations for 5 RNAs and the normal-mode perturbation method for 15 RNAs. For the MD dataset, there are 3,500 decoys for each of four RNAs whose RMSDs range from 0 to >10 Å, and 2,600 decoys for one RNA (PDB ID: 1msy) whose RMSDs range from 0 to 8 Å. Meanwhile, for the normal-mode dataset, there are about 490 decoys for each of the 15 RNAs, whose RMSDs range only from 0 to 5 Å. This dataset can be downloaded from http://csb.stanford.edu/rna. One point that should be noted is that the downloaded pdb files name atom O2 in pyrimidine bases as “O.”

Test dataset III comes from *RNA-Puzzles* rounds I to III [55–57], a collective and blind experiment in 3D RNA structure prediction. Given the nucleotide sequences, interested groups submit their predicted structures to the *RNA-Puzzles* website before the experimentally determined crystallographic or NMR structures of these target sequences are published. Therefore, the dataset is produced in a real RNA modeling scenario and can reveal the real performance of the existing scoring function. Marcin Magnus compiled the submitted structures from rounds I to III, and now the predicted models of 18 target RNAs can be downloaded from https://github.com/RNA-Puzzles/RNA-Puzzles-Normalized-submissions. There are only 12–70 predicted models for the 18 RNAs, some of whose RMSDs range from 2 to 4 Å, while some cover a wide range from 20 to 60 Å.

### Two trained neural networks

Two neural networks were trained based on two sets of training samples. The first set included only MD training samples and the second set included both MD and MC training samples. And the two network models are named RNA3DCNN_MD and RNA3DCNN_MDMC, respectively. We tested test datasets I and II using RNA3DCNN_MD, and tested test dataset III using RNA3DCNN_MDMC.

The reason why we trained two neural networks is that the three test datasets come from two kinds of methods. Test dataset I and II were produced by MD and normal-mode methods initiated from native structures, while test dataset III was produced by MC structure prediction methods, covering a broad structural space. After testing, for test datasets I and II, RNA3DCNN_MD performed better than RNA3DCNN_MDMC. But for test dataset III, RNA3DCNN_MDMC was superior. The results are reasonable. RNA3DCNN_MD is more accurate in the region close to native structures in that most of the MD training samples are not very far away from native structures or native topologies. However, when MC training samples were included, the neural network RNA3DCNN_MDMC became not as accurate as RNA3DCNN_MD for the structures around native ones and biased the non-native. On the contrary, RNA3DCNN_MD did not see the more random training structures far away from native states and thus it did not perform as well as RNA3DCNN_MDMC for test dataset III.

## Results and discussion

### Evaluation metrics

In general, a scoring function with good performance should be able to recognize the native structure from a pool of structural decoys and to rank near-native structures reasonably. Consequently, two metrics were used for a quantitative comparison with other scoring functions. One was the number of native RNAs with minimum scores in the test dataset, and the other was the Enrichment Score (*ES*) [34, 35, 58], which characterizes the degree of overlap between the structures of the top 10% scores (*E*_*top*10%_) and the best 10% RMSD values (*R*_*top*10%_) in the structural decoy dataset. The *ES* is defined as

$$\begin{array}{c} ES=\frac{|E_{top10\%}\cap R_{top10\%}|}{0.1\times 0.1\times N_{decoys}} \end{array}$$ (7)

where |*E*_*top*10%_ ∩ *R*_*top*10%_| is the number of structures in both the lowest 10% score range and the lowest 10% RMSD range, and *N*_*decoys*_ is the total number of structures in the decoy dataset. If the score and RMSD are perfectly linearly correlated, *ES* is equal to 10. If they are completely unrelated, *ES* is equal to 1. If *ES* is less than 1, the scoring function performs rather poorly with respect to that decoy dataset.

### Performance comparisons for test dataset I

We compared our CNN-based scoring function with four traditional statistical potentials for RNA, namely, 3dRNAscore, KB, RASP, and Rosetta.

**Fig 2:**
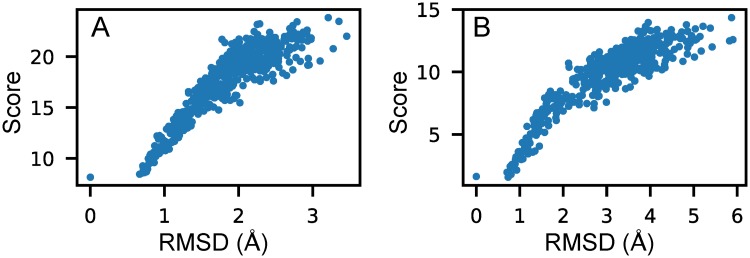
The relationship between RMSD and the score calculated by our method RNA3DCNN for two examples in test dataset I. (A) and (B) correspond to RNA 1y39D and 1q96C, respectively.

First, the number of native RNAs with minimum scores was counted as listed in Table 1. As the 3dRNAscore paper reported, 3dRNAscore identified 84 of 85 native structures, KB 80 of 85, RASP 79 of 85, and Rosetta 53 of 85. 3dRNAscore is thus clearly the best among the four statistical potentials. Our RNA3DCNN identified 62 of 85 native structures, and the unidentified native structures generally had the second or third lowest scores, almost the same as the lowest scores. Fig 2A shows an example in test dataset I in which the native structure was identified by our method, and Fig 2B shows an example in test dataset I in which the native structure had a slightly higher score calculated by our method than the structure of an RMSD of 0.9 Å. The RMSD-score plots of all 85 examples are provided in S1 Fig. The result that our method identified fewer native structures is reasonable. Specifically, the input and output of our neural network are geometry based, and thus similar structures have similar scores. The structures in the 0–1 Å range generally resemble each other and thus, for our scoring function, all the non-native structures with minimum scores have an RMSD ∼1 Å. Meanwhile, for the statistical potentials, atom steric clashes, angle, or dihedral angle deviations from the native form may quickly increase the potential values.

**Table 1:** The number of native structures with minimum scores calculated by our RNA3DCNN, 3dRNAscore, KB potential, RASP, and Rosetta methods in test dataset I to III.

	RNA3DCNN	3dRNAscore	KB	RASP	Rosetta
Test dataset I	62/85	84/85	80/85	79/85	53/85
Test dataset II MD	4/5	5/5	5/5	1/5	2/5
Test dataset II normal-mode	15/15	12/15	15/15	11/15	10/15
Test dataset III	13/18	5/18	—	1/18	4/18

Second, the *ES* was calculated. The mean *ES* values of the 85 RNAs calculated by 3dRNAscore, RASP, Rosetta, and our method RNA3DCNN were 8.69, 8.69, 6.7, and 8.61, respectively. The mean *ES* calculated by KB is not given in that we cannot open its original website and download its program, and the results of KB method shown in this paper come from the papers on KB and 3dRNAscore. The *ES* values of 3dRNAscore and our method are almost the same. The mean *ES* values of three methods are very large, suggesting that the RMSDs and scores calculated by the different methods are highly linearly correlated and that this test dataset is an easy benchmark to rank near-native decoys.

### Performance comparisons for test dataset II

**Fig 3:**
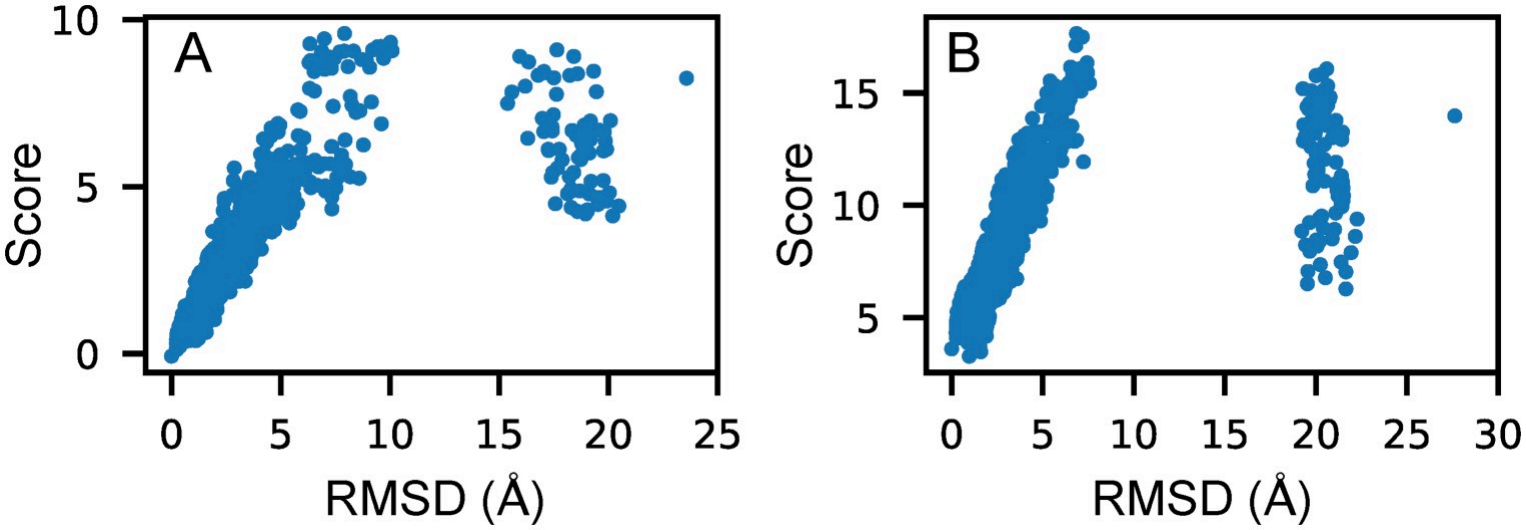
The relationship between RMSD and the score calculated by our method RNA3DCNN for two examples in test dataset II. (A) and (B) correspond to RNA 434d and 1f27, respectively.

For the MD decoys in test dataset II, 3dRNAscore and KB identified 5 of 5 native structures, RASP 1 of 5, Rosetta 2 of 5, and our method 4 of 5, as listed in Table 1. Our method gave the lowest score to the decoy of an RMSD of 0.97 Å for RNA 1f27, as shown in Fig 3B. The *ES* values of the MD decoys using different scoring functions are listed in Table 2. Fig 3A shows the relationship between RMSD and the score calculated by our method for the RNA 434d with the best *ES*. The RMSD-score plots of all five examples are provided in S2 Fig. From the table, we can see that our method performed better than 3dRNAscore for 2 of 5 RNAs, slightly worse for 1 of 5 RNAs, and worse for 2 of 5 RNAs, especially for the RNA 1f27, in that the native structure had a slightly higher score than the decoys of RMSD around 1 Å. Moreover, our method performed better than KB, RASP, and Rosetta for 3 of 5 RNAs, comparably for 1 of 5 RNAs, and worse for the RNA 1f27, as explained above.

**Table 2:** The *ES* values of our RNA3DCNN, 3dRNAscore, KB potential, RASP, and Rosetta methods in test dataset II.

Decoy generation method	RNA	Length	RNA3DCNN	3dRNAscore	KB	RASP	Rosetta
MD	1duq	26	7.3	8.3	7.6	7.6	7.1
	1f27	30	4.2	8.2	7.9	6.7	6.2
	1msy	27	7.2	7.8	5.7	5.7	3.6
	1nuj	24	8.3	7.2	7.3	5.0	6.9
	434d	14	8.5	7.7	7.7	7.0	6.8
Mean values			7.1	7.8	7.2	6.4	6.1
Normal-mode	1duq	26	6.1	7.3	7.0	5.7	3.8
	1esy	19	7.1	4.9	5.4	4.3	5.6
	1f27	30	6.1	6.1	5.8	3.7	2.6
	1i9v	76	5.7	5.3	2.6	5.5	3.0
	1kka	17	3.3	6.1	4.6	3.9	4.6
	1msy	27	6.9	6.1	5.6	2.2	4.6
	1nuj	24	7.1	7.1	7.4	6.1	2.4
	1qwa	21	4.5	3.9	3.2	2.0	3.8
	1x9k	62	5.2	5.2	1.6	5.4	3.0
	1xjr	46	5.6	7.3	5.4	7.7	2.2
	1ykq	49	5.4	4.8	3.4	3.5	2.8
	1zih	12	5.5	6.9	5.4	5.7	6.6
	28sp	28	6.7	5.1	4.0	6.7	1.8
	2f88	34	6.3	6.3	5.4	4.8	4.4
	434d	14	7.8	7.3	7.4	7.3	5.2
Mean values			6.0	6.0	4.9	5.0	3.8

For the normal-mode decoys in this dataset, 3dRNAscore identified 12 of 15 native structures, RASP 11 of 15, Rosetta 10 of 15, KB and our method 15 of 15, as listed in Table 1. The *ES* values of the normal-mode decoys using different scoring functions are also listed in Table 2. From the table, we can see that our method performed better than 3dRNAscore for 7 of 15 RNAs, equally for 4 of 15 RNAs, and worse for only 4 of 15 RNAs. Moreover, our method performed better than KB, RASP, and Rosetta for 12, 11, and 13 of 15 RNAs. The mean *ES* values of 3dRNAscore and our method were the same, and were greater than the other scoring functions. The RMSD-score plots of all 15 examples are provided in S2 Fig.

### Performance comparisons for test dataset III

**Fig 4:**
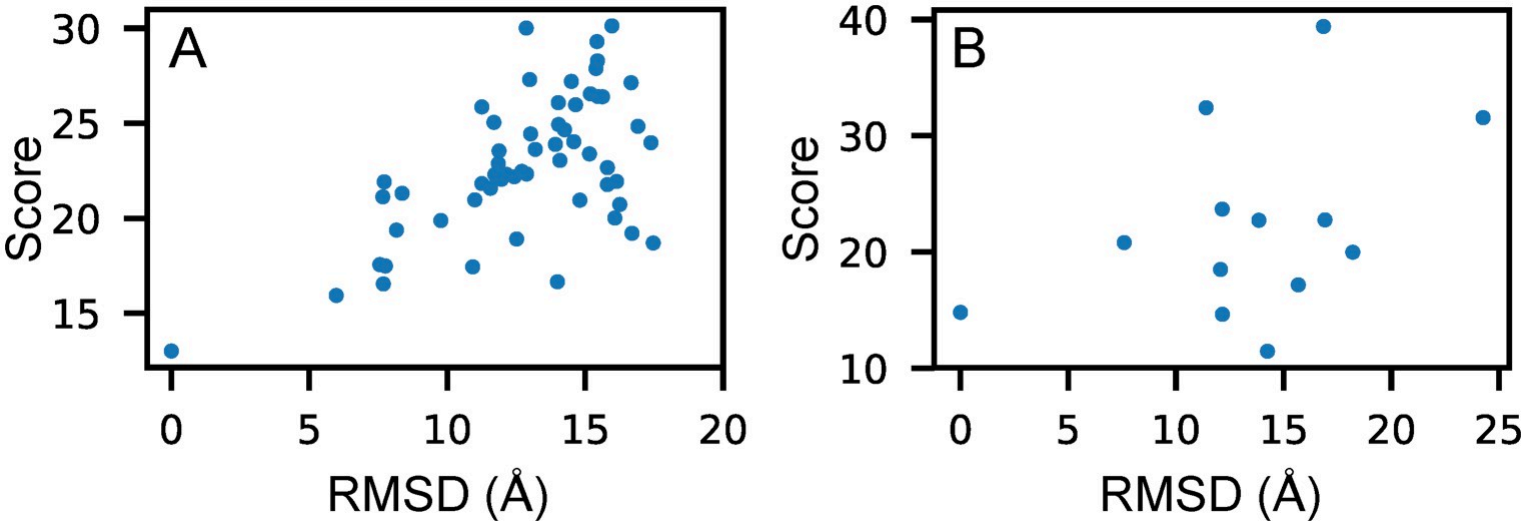
The relationship between RMSD and the score calculated by our method RNA3DCNN for two examples in test dataset III. (A) and (B) correspond to Puzzle-14-Bound and Puzzle-3, respectively.

The structures in test dataset III are derived from different groups by different RNA modeling methods. There are only dozens of predicted models for each target RNA and the RMSDs are almost always greater than 10 Å, and often even greater than 20, or 30 Å. Consequently, we did not calculate the *ES* for this dataset and gave only the RMSDs of models with minimum scores in Table 3. The results of method KB were not provided in that we could not open its website and get the program. From the table, we can see that our RNA3DCNN identified 13 of 18 native RNAs, 3dRNAscore 5 of 18, RASP 1 of 18, and Rosetta 4 of 18. For puzzle 2, though the native structures were not identified, our method gave the lowest RMSD among four methods. And for puzzle 3, our method gave the RMSD as low as other two methods. Fig 4A shows an example in test dataset III in which the native structure was well identified by our method, and Fig 4B is the one not identified. The RMSD-score plots of all 18 examples are provided in S3 Fig.

**Table 3:** The RMSDs of structures with minimum scores calculated by our RNA3DCNN, 3dRNAscore, RASP, and Rosetta methods in test dataset III.

Puzzle-X	Length	RNA3DCNN (Å)	3dRNAscore (Å)	RASP (Å)	Rosetta (Å)
1	46	0.0	0.0	5.7	4.5
2	100	2.3	3.7	3.7	3.5
3	84	14.3	14.3	14.3	15.7
4	126	4.5	4.2	4.2	4.5
5	188	0.0	0.0	0.0	9.8
6	168	0.0	29.0	30.9	13.5
7	185	0.0	0.0	42.5	20.6
8	96	0.0	0.0	10.7	0.0
10	171	10.4	10.3	9.3	6.8
12	125	0.0	13.2	13.2	13.0
13	71	0.0	0.0	15.2	5.4
14-Bound	61	0.0	6.0	7.6	11.7
14-Free	61	0.0	11.4	11.4	9.8
15	68	0.0	19.8	16.3	8.7
17	62	0.0	8.6	15.1	0.0
18	71	0.0	16.4	12.5	5.6
19	62	16.5	16.5	16.5	0.0
21	41	0.0	18.0	18.0	0.0

For test datasets I and II, all decoys are obtained from native structures, which means that they almost always stay around one local minimum in the energy landscape. But for test dataset III, in the real modeling scenario, the structures are far from native topologies and are located at different local minima in the energy landscape. For this reason, we trained two neural networks with two sets of training samples, that is, one set including only training samples from MD simulations initiated from native structures and another set including both MD training samples and MC training samples obtained in the broader and more complicated structural space.

### Nucleotide quality assessment

**Fig 5:**
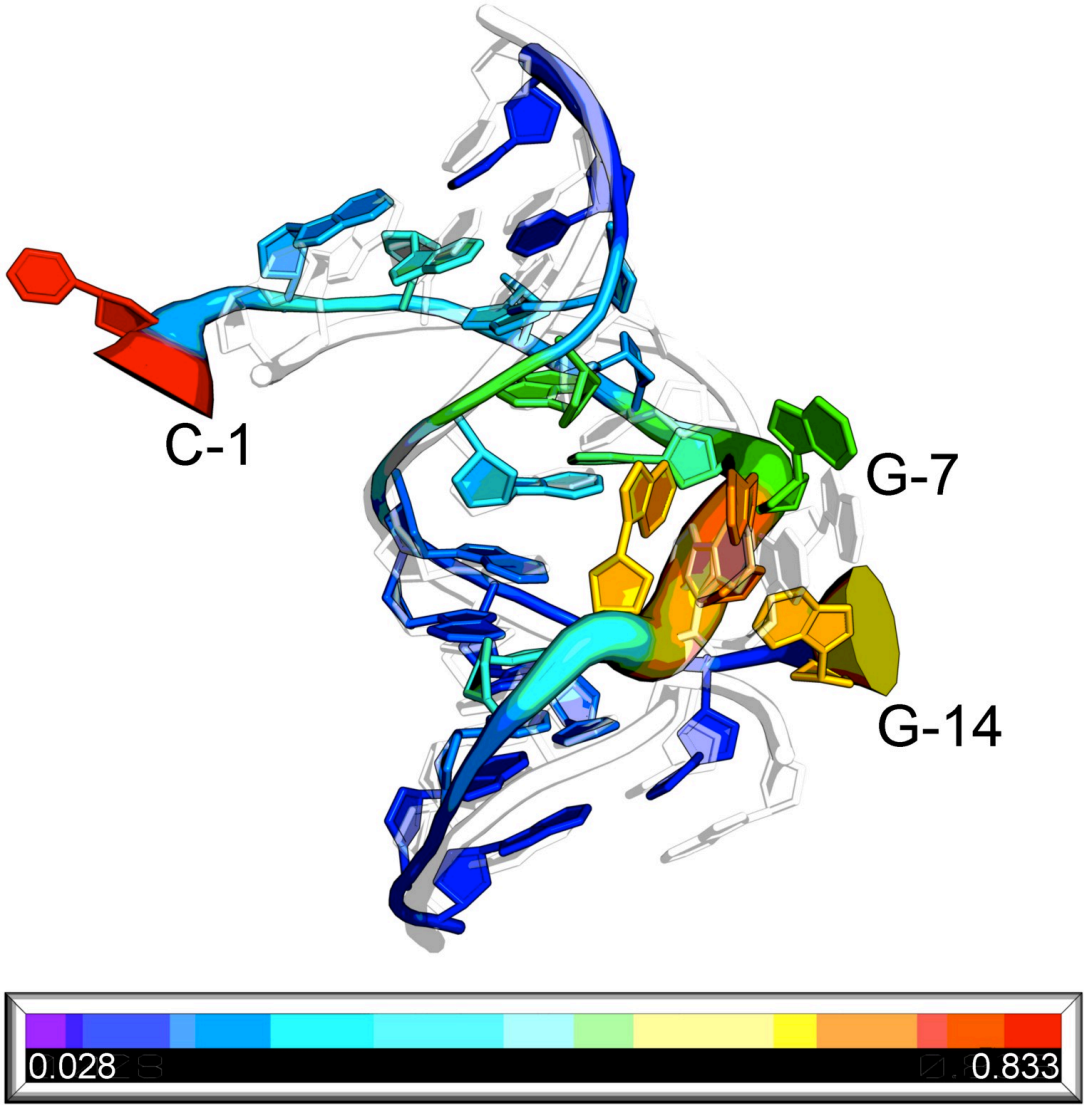
Nucleotide quality assessment. The colorful cartoon is a structural decoy of an RMSD of 3.0 Å with respect to its native structure RNA 1nuj drawn as a white semi-transparent cartoon. The thickness of the colorful tube represents the RMSD of each nucleotide from its native counterpart after optimal superposition between the decoy and the native structure. A thicker tube means a larger RMSD. The rainbow colors represent the nucleotide unfitness scores calculated by our RNA3DCNN method. From blue to red, the unfitness scores increase.

Our scoring function can evaluate each nucleotide, reveal the regions in need of further structural optimization, and guide the sampling direction in RNA tertiary structure modeling. Fig 5 portrays how our scoring function helps locate the unfit regions. In this figure, a decoy of RMSD 3.0 Å from test dataset II MD decoys and the native RNA 1nuj are superimposed, and thicker tubes show larger deviations from the native structure. The rainbow colors represent the calculated unfitness scores of each nucleotide, and the colors closer to red represent larger unfitness scores. We can see that the tubes in nucleotides 1, 7, 8, 9, and 14 are much thicker, and the colors of those regions are much closer to red, which means that our scoring function can rank the nucleotide quality correctly. Nucleotides 1 and 14 are the terminal nucleotides in two chains and are unpaired, so the deviations of these two are the largest. Nucleotides 7–9 are in the internal loop, so the deviations are larger than those of the remaining helical regions.

The Pearson correlation coefficients between actual and predicted nucleotide unfitness scores were 0.69 and 0.34 for MD decoys and NM decoys in test dataset II, respectively, as shown in S4 Fig. The structures in NM decoys are all near native structures with RMSD ranging from 0 to 5 Å, thus making the correlation not strong.

### Network visualization: Saliency maps

Saliency maps were used to visualize the trained network and help understand which input atoms are important in deciding the final output. In paper [59], an image-specific class saliency map was first introduced to rank the pixels of an input 2D image based on their influence on the class score by computing the gradient of output class score with respect to the input image. The gradient can reveal how sensitive the class score is to a small change in input image pixels. Larger positive gradients mean that a slight decrease in the corresponding pixels can cause the true class score to drop markedly, and thus the corresponding pixels are more important in determining the right output class. Meanwhile, for our regression problem and a near-native conformation, the smaller output was better and the voxels of negative gradients were highlighted and important. Moreover, we mapped the gradients of each voxel back to the corresponding atoms.

**Fig 6:**
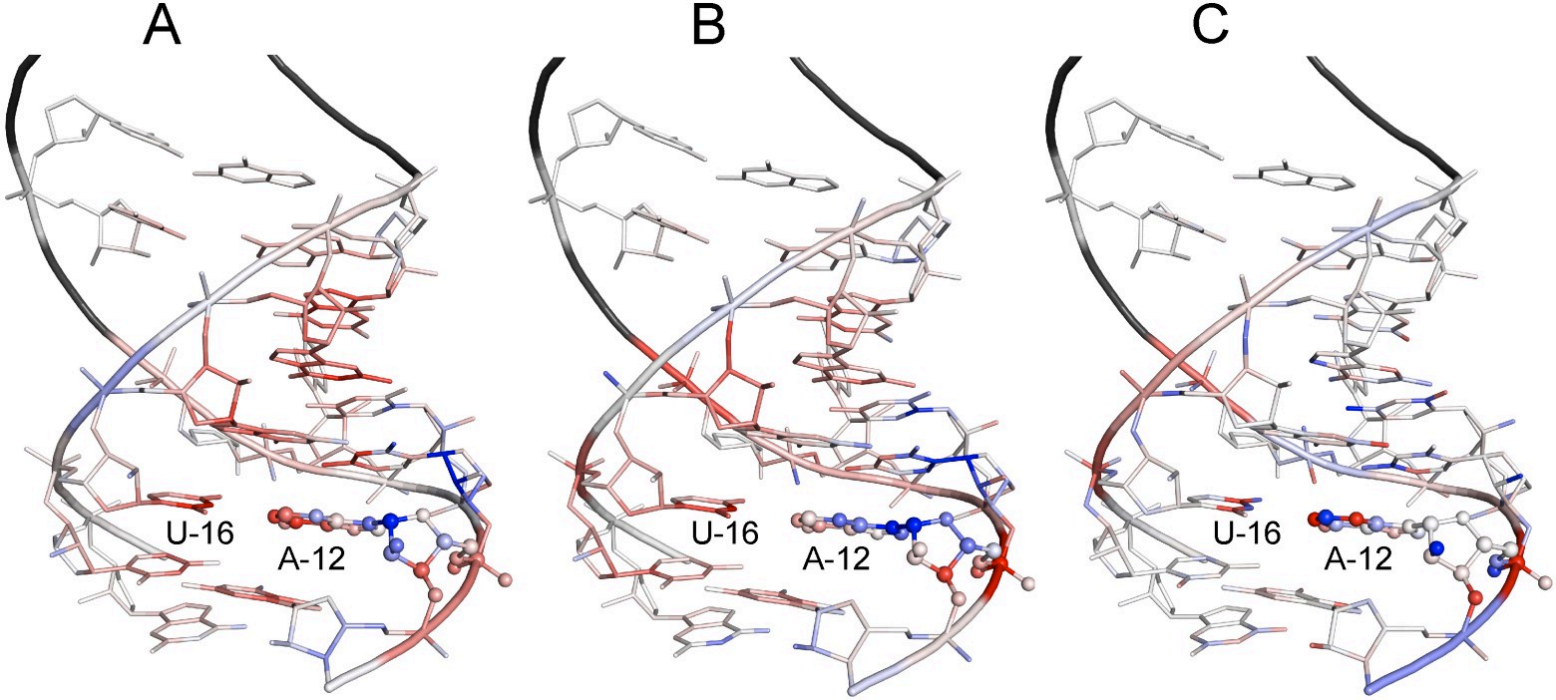
Saliency maps. (A), (B), and (C) are the saliency maps for the three input channels, namely, atomic occupation number, mass, and charge channels, respectively. The 12th nucleotide of the native RNA 1nuj under assessment is drawn as spheres and sticks, its surrounding environment is drawn as sticks, while the atoms left are drawn as a black cartoon. The redder atoms represent smaller negative gradients, the bluer atoms represent larger positive gradients, and the nearly white atoms represent gradients close to 0. The red atoms are highlighted and important in deciding the final output.

In Fig 6, examples of saliency maps for the three input channels are presented. A, B, and C correspond to atomic occupation number, mass, and charge channels, respectively. The example is used to calculate the unfitness score of the 12th nucleotide in a helical region for the native RNA 1nuj. The nucleotide under assessment is drawn as spheres and sticks, its surrounding environment is drawn as sticks, while the atoms beyond its surrounding environment are shown as a black cartoon. The redder atoms represent smaller negative gradients, the bluer atoms represent larger positive gradients, and the nearly white atoms represent gradients close to 0. The red regions are highlighted and more important in deciding the final output. In the atomic occupation number channel, atomic category differences disappear and only shapes count. From Fig 6A, we can see that the atoms in the nucleobases of the 10th–13th and 15th–19th nucleotides are highlighted and atom N3 in the 16th nucleotide is the most important, in accordance with the base-pairing and base-stacking interactions. In the atomic mass channel, the importance of atoms in the nucleobases described above declines somewhat, while atom P in the 12th nucleotide and atom N3 in the 16th nucleotide are the most important, in that atom P is much heavier than atoms C, N, and O and atom N3 is in the A12’s paired-base U16. In the atomic charge channel, the seven most important atoms are N1, P, N3, and O3’ in the 12th nucleotide, atoms C4 and C2 in the 16th nucleobase, and atom N2 in the 17th nucleobase. Overall, from the analyses of the salient maps, it was found that the neural networks can learn the knowledge, such as the relevance of base pairing and stacking interactions to the score, from the training data automatically without any priori knowledge. It would be very interesting to see if neural networks can dig new knowledge out of data in the future work.

### The computational speed

We tested the computational time of 100 decoys of 91 nucleotides. The total time was 321.0 seconds. For a comparison, the C++ version of 3dRNAscore method took only 19 seconds. However, it was found that 99.6% of our computational time (319.7 seconds) was used to prepare the input to CNN, and this time decreased to 2 seconds after we changed the code from Python to C++. Therefore, the CNN-based approach is very efficient in terms of speed, and it is estimated that the overall computational time of our method will be approximately 3 seconds if we rewrite the entire code in C++. However, the computational time of our method in Python version is acceptable for now, at least temporarily. We postpone the code rewriting work to the future when necessary. Moreover, our method can be downloaded from https://github.com/lijunRNA/RNA3DCNN.

### Conclusion

Recently, we have witnessed the astonishing power of machine learning methods in characterizing, classifying, and generating complex data in various fields. It is therefore interesting to explore the potential of machine learning in characterizing and classifying RNA structural data. In this study, we developed two 3D CNN-based scoring models, named RNA3DCNN_MD and RNA3DCNN_MDMC, for assessing structural candidates built by two kinds of methods. If the structural candidates are generated by MC methods such as fragment assembly, RNA3DCNN_MDMC is suggested. If the structural candidates are not very far away from the native structures, such as from MD simulations, the RNA3DCNN_MD model is better. We also compared our method with four other traditional scoring functions on three test datasets. The current 3D CNN-based approaches performed comparably with or better than the best statistical potential 3dRNAscore on different test datasets. For the first test dataset, the mean *ES* was almost the same as that of the best traditional scoring function, 3dRNAscore. The reason why the number of native structures identified by our method was much smaller than that by other scoring functions is that our method is structure-based and the scores of native structures and decoys of RMSD less than 1.0 Å are almost the same. This suggests that our method is robust if an RNA structure does not change much. For the second test dataset, our method generally performed similarly to 3dRNAscore and outperformed the three other scoring functions. For the MD decoys in the second test dataset, our method was slightly worse than 3dRNAscore. For the normal-mode decoys in the second test dataset, our method identified all the native structures, while 3dRNAscore identified only 12 of 15 native RNAs, and our method outperformed 3dRNAscore for 7 of 15 RNAs and underperformed it for only 4 of 15 RNAs. For the third test dataset from blind and real RNA modeling experiments, our method was far superior to the other scoring functions in identifying the native structures.

Our method has some novel features. First, it is free of the choice of the reference state, which is a difficult problem in traditional statistical potentials. Second, it treats a cube of atoms as a unit like a many-body potential, while traditional statistical potentials divide them into atom pairs. Moreover, our method can evaluate each nucleotide, reveal the regions in need of further structural optimization, and guide the sampling direction in RNA tertiary structure prediction.

Our method demonstrates the power of CNNs in quality assessments of RNA 3D structures and shows the potential to far outperform traditional statistical potentials. There remains great scope to improve the CNN models, such as by expanding them to include more input channels (only three are considered currently), featuring more complex network architecture, and involving larger training datasets. Moreover, more RNA-related problems can be dealt with by 3D CNNs, such as protein–RNA binding affinity prediction and RNA–ligand docking and virtual screening.

## Supporting information

S1 Fig The relationship between RMSD and the score calculated by our method RNA3DCNN for 85 examples in test dataset I. (PDF)

S2 Fig The relationship between RMSD and the score calculated by our method RNA3DCNN for 20 examples in test dataset II. (PDF)

S3 Fig The relationship between RMSD and the score calculated by our method RNA3DCNN for 18 examples in test dataset III. (PDF)

S4 Fig The relationship between actual and predicted nucleotide unfitness scores for MD decoys and NM decoys in test dataset II. (PDF)

S1 Table The charge and mass information of each type of atoms extracted from amber03 force field in software Gromacs. (PDF)
