# Supplementary figures and images for: RNA3DCNN: Local and global quality assessments of RNA 3D structures using 3D deep convolutional neural networks
Source: PLoS Comput Biol. 2018 Nov 27;14(11):e1006514. doi: 10.1371/journal.pcbi.1006514 (PMC6258470; doi:10.1371/journal.pcbi.1006514)

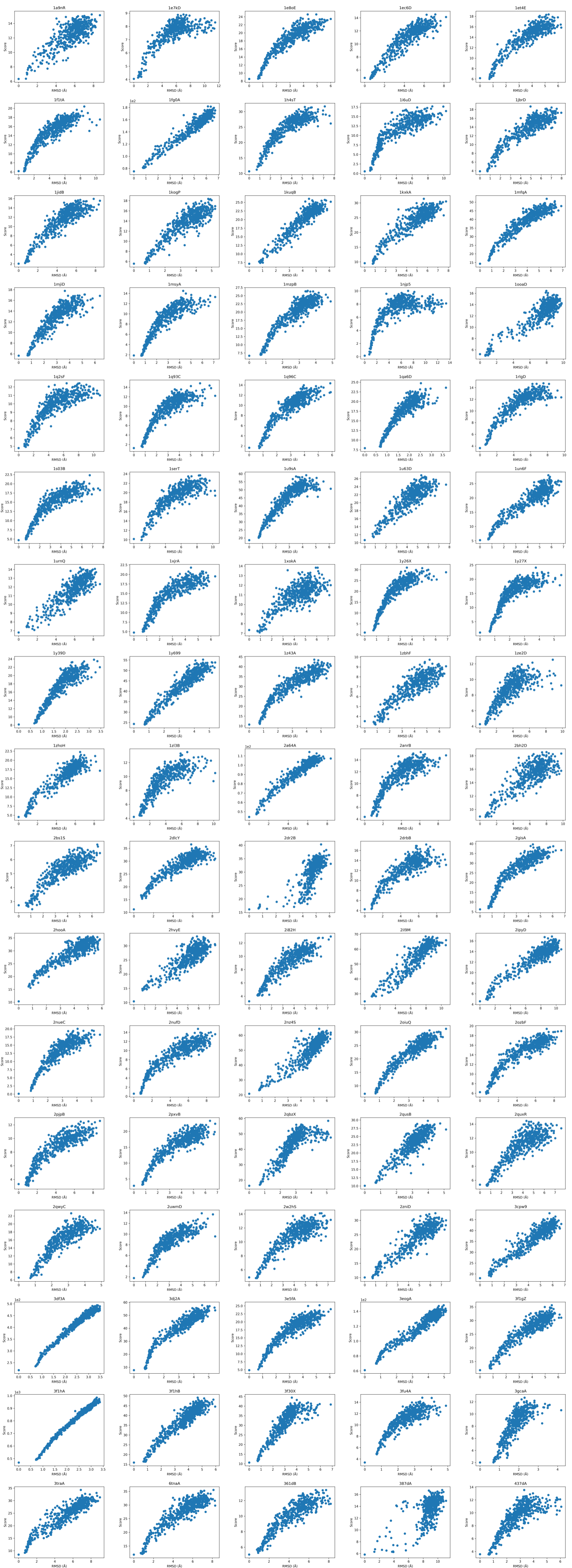

Supplement: S1 Fig — (PDF) [file pcbi.1006514.s001.pdf]

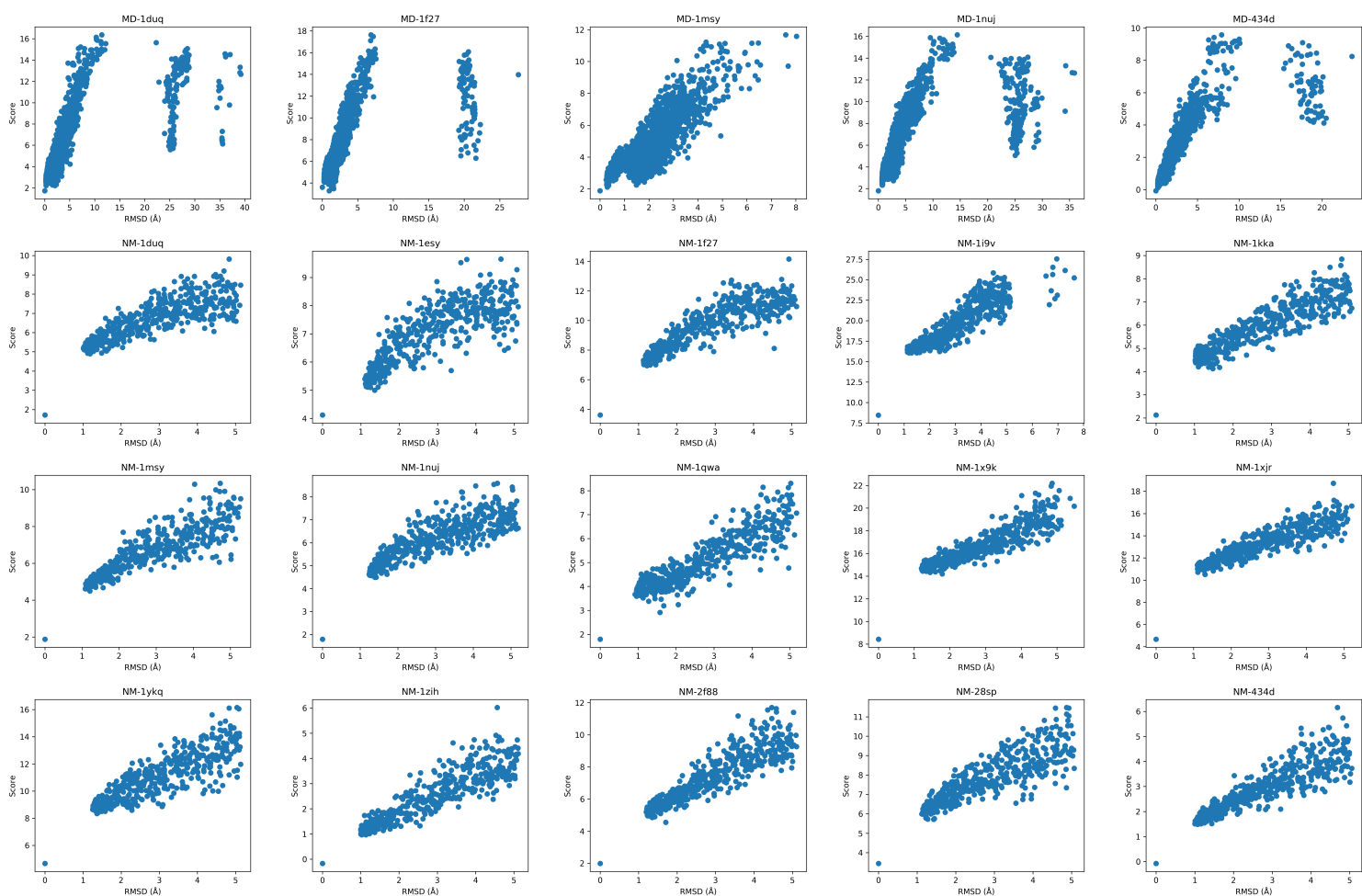

Supplement: S2 Fig — (PDF) [file pcbi.1006514.s002.pdf]

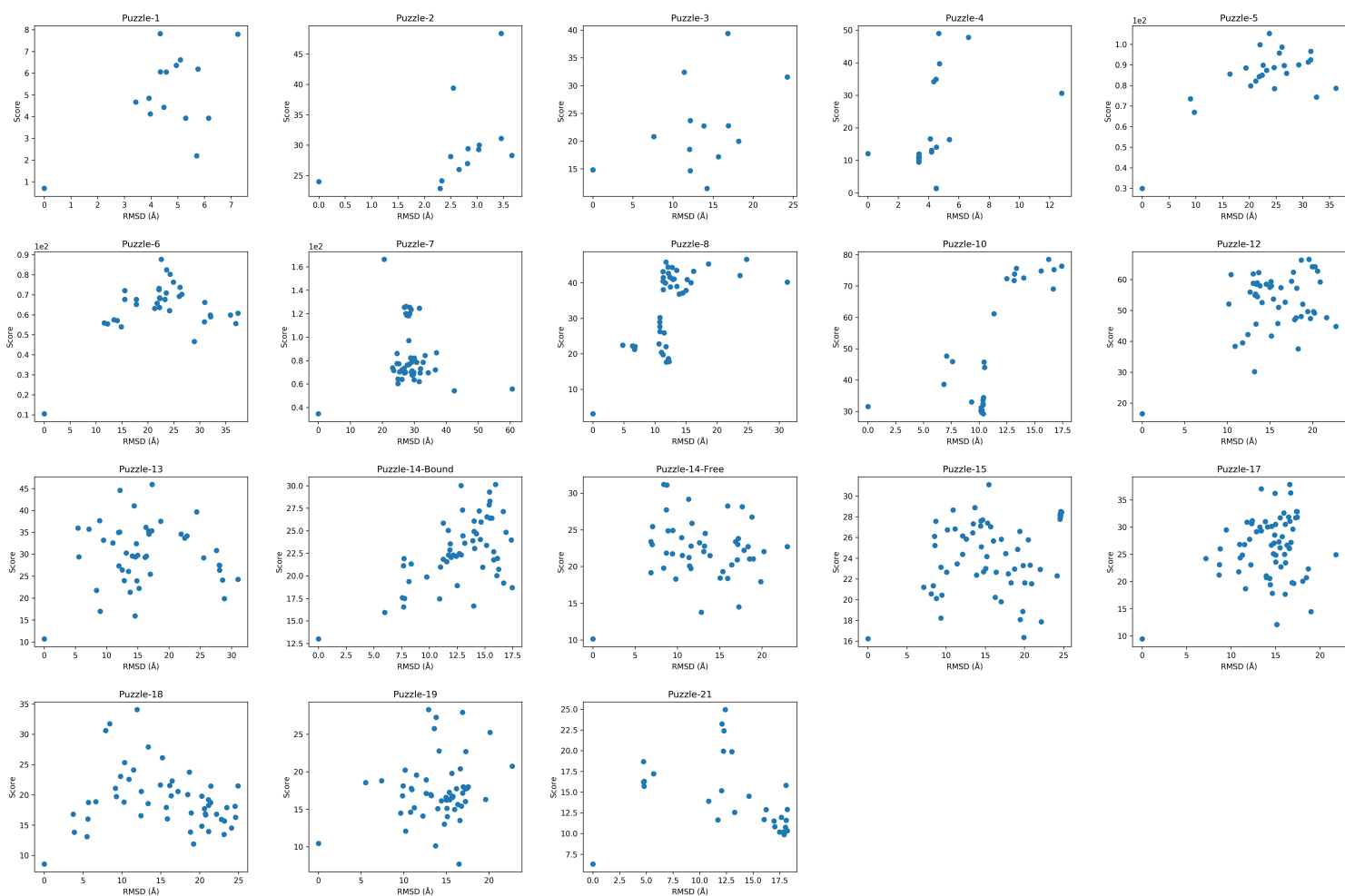

Supplement: S3 Fig — (PDF) [file pcbi.1006514.s003.pdf]

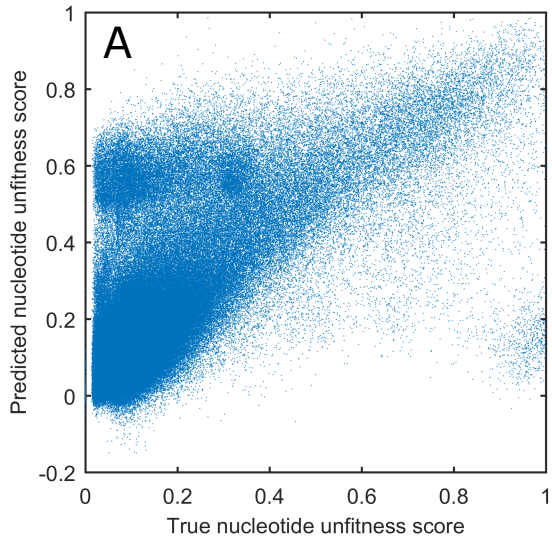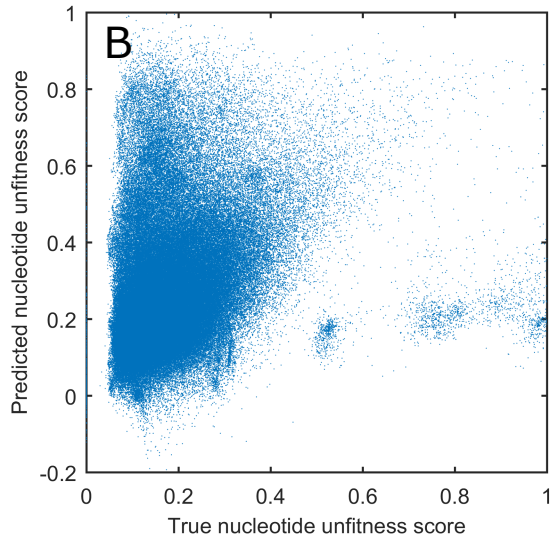

Supplement: S4 Fig — (PDF) [file pcbi.1006514.s004.pdf]
