# Supplementary material for: RNA3DCNN: Local and global quality assessments of RNA 3D structures using 3D deep convolutional neural networks
Source: PLoS Comput Biol. 2018 Nov 27;14(11):e1006514. doi: 10.1371/journal.pcbi.1006514 (PMC6258470; doi:10.1371/journal.pcbi.1006514)
Supplement: S1 Table — (PDF) [file pcbi.1006514.s005.pdf]

charge	A	G	U	C
P	1.1662	1.1662	1.1662	1.1662
OP1/OP2	-0.776	-0.776	-0.776	-0.776
O5’	-0.4989<br>(not in 5’-end)<br>0.6223<br>(in 5’-end)	-0.4989<br>(not in 5’-end)<br>0.6223<br>(in 5’-end)	-0.4989<br>(not in 5’-end)<br>0.6223<br>(in 5’-end)	-0.4989<br>(not in 5’-end)<br>0.6223<br>(in 5’-end)
C5’	0.0558	0.0558	0.0558	0.0558
C4’	0.1065	0.1065	0.1065	0.1065
C3’	0.2022	0.2022	0.2022	0.2022
O3’	-0.5246<br>(not in 3’-end)<br>-0.6541<br>(in 3’-end)	-0.5246<br>(not in 3’-end)<br>-0.6541<br>(in 3’-end)	-0.5246<br>(not in 3’-end)<br>-0.6541<br>(in 3’-end)	-0.5246<br>(not in 3’-end)<br>-0.6541<br>(in 3’-end)
O4’	-0.3548	-0.3548	-0.3548	-0.3548
C1’	0.0394	0.0191	0.0674	0.0066
C2’	0.067	0.067	0.067	0.067
O2’	-0.6139	-0.6139	-0.6139	-0.6139
N9	-0.0251	0.0492		
C8	0.2006	0.1374		
N7	-0.6073	-0.5709		
C5	0.0515	0.1744	-0.3635	-0.5215
C6	0.7009	0.477	-0.1126	0.0053
O6		-0.5597		
N1	-0.7615	-0.4787	0.0418	-0.0484
C2	0.5875	0.7657	0.4687	0.7538
N2		-0.9672		
N3	-0.6997	-0.6323	-0.3549	-0.7584
C4	0.3053	0.1222	0.5952	0.8185
N6	-0.9019			
O4			-0.5761	
O2			-0.5477	-0.6252
N4				-0.953

atom	mass
P	30.9738
O	15.9994
N	14.0067
C	12.0107
